# Manipulating the carrier concentration and phase transition via Nb content in SrTiO_3_

**DOI:** 10.1038/s41598-021-03199-7

**Published:** 2022-02-15

**Authors:** Zhe Zhang, Peihua Qian, Xingming Yang, Baixi Wu, H. L. Cai, F. M. Zhang, X. S. Wu

**Affiliations:** 1 Institute of Materials Engineering, Nanjing University, Nantong, Jiangsu 226019 China; 2 National Laboratory of Solid State Microstructures & Department of Physics, Nanjing University, Nanjing, 210093 China

**Keywords:** Electronic properties and materials, Phase transitions and critical phenomena

## Abstract

SrTiO_3_ is a model of the perovskite-like compounds for structural transition which inducing the intriguing physical properties around the critical phase transition temperature T_AFD_ (antiferrodistortive, abbrev. as AFD). Here we report that the electrical transport behavior is a new way to quantify Nb concentration for Nb-doped SrTiO_3_. The lattice parameter (c), phase transition temperature (T_AFD_), and the carrier concentration (n) of SrTiO_3_ may be manipulated by niobium doping. T_AFD_ increases with increasing the niobium content in a rate of about 30 K per (wt%, i.e. niobium element’s weight verses total weight) niobium and n in a rate of about 2.5 $\times$ 10^20^/cm^3^ per (wt%) niobium.

## Introduction

Strontium titanate (SrTiO_3_, abbrev. as STO) is a well known as one of the typical perovskite-like oxides with the general formula ABO_3_. The STO system has attracted considerable attentions not only for being a model transition-metal oxide system where fundamental physics has been extensively studied^1–3^, but also for its potential application in new device fabrications due to its tunable electronic, optical and transport properties^4–12^. Many interesting properties have been discovered in the STO system, for example: STO is a quantum paraelectric^13^ as well as the first reported insulating oxide superconducting material^14^; LaAlO_3_/SrTiO_3_ heterointerface has two-dimensional electron gas with highly mobility of charge carriers^15,16^; migration of oxygen vacancies occurs at BaTiO_3−δ_/SrTiO_3_ interface^17^; ferromagnetic metallic phase and antiferromagnetic insulating phase occur in strained SrRuO_3_/SrTiO_3_ superlattices^18^. However, technology application requires manipulating the physical properties.

Doping is one of the easiest and most efficient ways to manipulate physical properties of STO, not only because of the change of the carriers, but also the structural change of the unit cell^19–21^. Doping of a small electrons may raise abundant interaction among charge, spin, orbital and lattice degree of freedom for SrTiO_3_, therefore display the intriguing physical properties such as rectifying behavior^22^, resistive switching phenomenon^4,9^, ionic polarization^23^, high permittivity with low dielectric loss^24,25^, thermoelectric^26,27^, inverse spin Hall effect^28^, luminescence^29^, quantum ferroelectric^30^ and so on. Furthermore, many of these physical properties are believed to alter around the critical phase transition temperature (T_AFD_ = 105 K, antiferrodistortive, abbrev. as AFD) of STO. The essential character of this phase transition is that when temperature goes down under T_AFD_, the TiO_6_ octahedral of STO rotate around the c-axis in an antiferrodistortive pattern driven by softening of a Brillouin zone boundary phonon^31^. Studies have shown that this soft mode and the AFD transition affect the specific heat^32^, thermal conductivity^33^, sound velocity^34^, thermal expansion^35^ and conductivity anisotropy^20^ of STO tremendously. Hence, to clarify the relationship between doping concentration and electrical transport property around T_AFD_ is very important for us to understand the impact of the phase transition on electronic transport.

In the present, (001)-oriented Nb-doped SrTiO_3_ single crystals with Nb doping concentration of 0, 0.05%, 0.1%, 0.5%, and 0.7% (wt%) are grown and studied. Results show that Nb doping, as well as the further post-annealing may be a way to revise the structure and properties of STO single crystals. We observe the variation of electrical transport behavior around T_AFD_. The phase transition temperature and the carrier concentration of Nb-doped SrTiO_3_ vary linearly with the Nb concentration, which is very meaningful for understanding the physics of the future application in electrical devices.

## Methods

(001)-oriented Nb-doped SrTiO_3_ single-crystal slices with varying the mass percent (wt%:0, 0.05%, 0.1%, 0.5%, 0.7%, i.e. niobium element’s weight verses total weight; nominal formulas are SrTiO_3_, SrTi_0.999_Nb_0.001_O_3_, SrTi_0.998_Nb_0.002_O_3_, SrTi_0.990_Nb_0.010_O_3_ and SrTi_0.986_Nb_0.014_O_3_ respectively) of Nb are prepared and polished. Stoichiometric amounts of SrCO_3_, TiO_2_ and Nb_2_O_5_ were mixed and then grew by flame fusion method basically following Ref.^36^, the growth speed was 15 mm/h and under an atmosphere of H_2_/O_2_ mixture with the H_2_/O_2_ ratio of 5:1. Nb-free, and 0.5% Nb-doped SrTiO_3_ single crystals are further annealed in a tube furnace in air at the temperature 930 °C for more 2 h, for comparison after the crystals remain in air for more than one year. The X-ray diffraction data and rocking curve data are obtained on the beamline BL14B1 located at the Shanghai Synchrotron Radiation Facility (SSRF), and the RSM (reciprocal space mapping) data near the corresponding symmetric 002 reflection is also obtained using X-ray with a wavelength of 1.2387 Å. The detailed information about the beamline BL14B1 following Yang et al.^37^. Raman spectra measurement is carried out using micro-Raman spectrometer LabRAM HR800 equipped with a low temperature platform. A 488 nm laser is selected as the excitation source. An objective × 50 is used both to focus the incident laser beam and to collect the scattered light. Transport property is characterized by PPMS (Physical Property Measurement System) from Quantum Design (PPMS-9).

## Results and discussion

**Figure 1:**
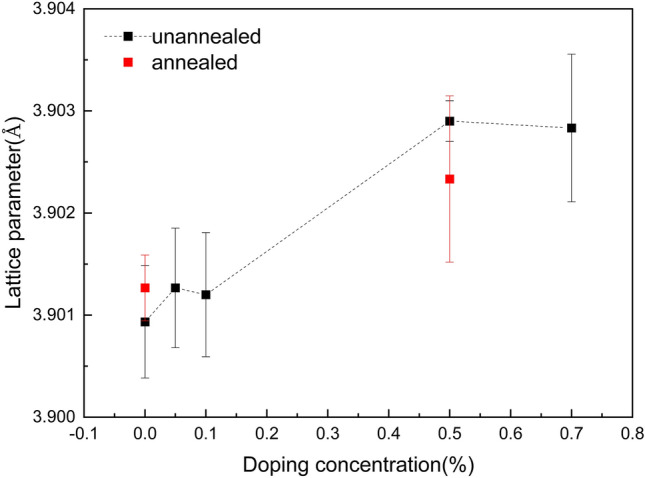
The out of plane lattice parameter c dependence of Nb-doping concentration in SrTiO_3_ crystals.

X-ray diffraction reflections around the (001), (002) and (003) diffraction peaks are fitted using a Gaussian Function, and calculate the out of plane lattice parameter c based on Bragg equation. All FWHMs are small enough (around 0.01°–0.02° which is very close to the resolution of the 6-cycle diffractometer which is 0.01°), indicating that the single crystals here have high quality. There are clear yet small (less than 0.05°) peak shift as Nb element is doped into STO. Figure 1 shows the calculated out of plane lattice parameter c of Nb doped SrTiO_3_ single-crystals. Post annealing for sample with doping free and 0.5%Nb doping SrTiO_3_ shows that the lattice symmetry remains the same the ones before annealing. Whiles, after annealing the lattice parameter c for Nb-free STO increases, and for Nb-doped STO decreases, as shown in Fig. 1. We argue that Nb^5+^ ions (0.64 Å) have bigger ionic radius than Ti^4+^ (0.605 Å), and induces the lattice increase with increasing the Nb concentration. In addition, more Nb doped might leads to more Ti^3+^ ions (0.67 Å) whose ionic radius is also bigger than Ti^4+^ ions (0.605 Å), which caused the lattice expansion^38,39^. In addition, some defects, inclusion oxygen vacancy, anti-site occupying between Sr and Ti, may occur during crystal growth by flame fusion, which may be recovered during the further annealing (at least around the near surface part due to the small velocity of the transfer atoms in the crystal). We believe that the lattice parameter of c increases slightly after annealing for Nb-free STO tells the recover the truth. Nb doping may lead to stacking fault and dislocation fault, which may increase the calculated average parameter c.

We performed rocking curve measurement to try to calculate the density of the misfit dislocation fault densities (MD)^40^. For a given reflection, the measured rocking curve full width at half maximum (FWHM) *β*_m_ including the intrinsic half width *β*_i_ for the perfect sample is given by:

1 $$\upbeta _{{\text{m}}}^{2} = \upbeta _{{\text{i}}}^{2} + \upbeta _{{\text{d}}}^{2} + \upbeta _{\upvarepsilon }^{2} + \upbeta _{\upalpha }^{2} + \upbeta _{{\text{L}}}^{2} + \upbeta _{{\text{r}}}^{2} .$$

The intrinsic rocking curve width for the crystal is usually less than several tens of arcseconds and can often be neglected^41^. *β*_d_ is the intrinsic rocking curve width from the diffractometer; *β*_*ε*_, *β*_*α*_, *β*_L_ and *β*_r_ are the rocking curve broadening caused by the strain surrounding dislocations, lattice tilting, particle size and curvature, respectively. A simple model^42^ gives the dislocation density as:

2 $${D}_{dis}\approx {\left(\Delta \beta \right)}^{2}/9{b}^{2},$$

where *D*_dis_ is the MD, *b* is the length of the Burger vector of the corresponding dislocation and ${\left(\Delta \beta \right)}^{2}={{\upbeta }_{\text{m}}}^{2}-{{\upbeta }_{\text{i}}}^{2}-{{\upbeta }_{\text{d}}}^{2}$.

According to our calculation result, the total dislocation densities of our samples are of the order of about 10^7^ cm^−2^, which is smaller than films fabricate by laser molecular beam epitaxy^40^. For comparison, the DM among the annealed samples is around 10% smaller than other samples.

As for the reason why annealing process leads to a decrease of lattice parameter of doped sample, we believe that annealing process may erase these stacking fault and dislocation fault, and also distortion due to the non-chemical stoichiometric, crystal become more quality, which may be proved by the following reciprocal space mapping (RSM) measurements^43^.

**Figure 2:**
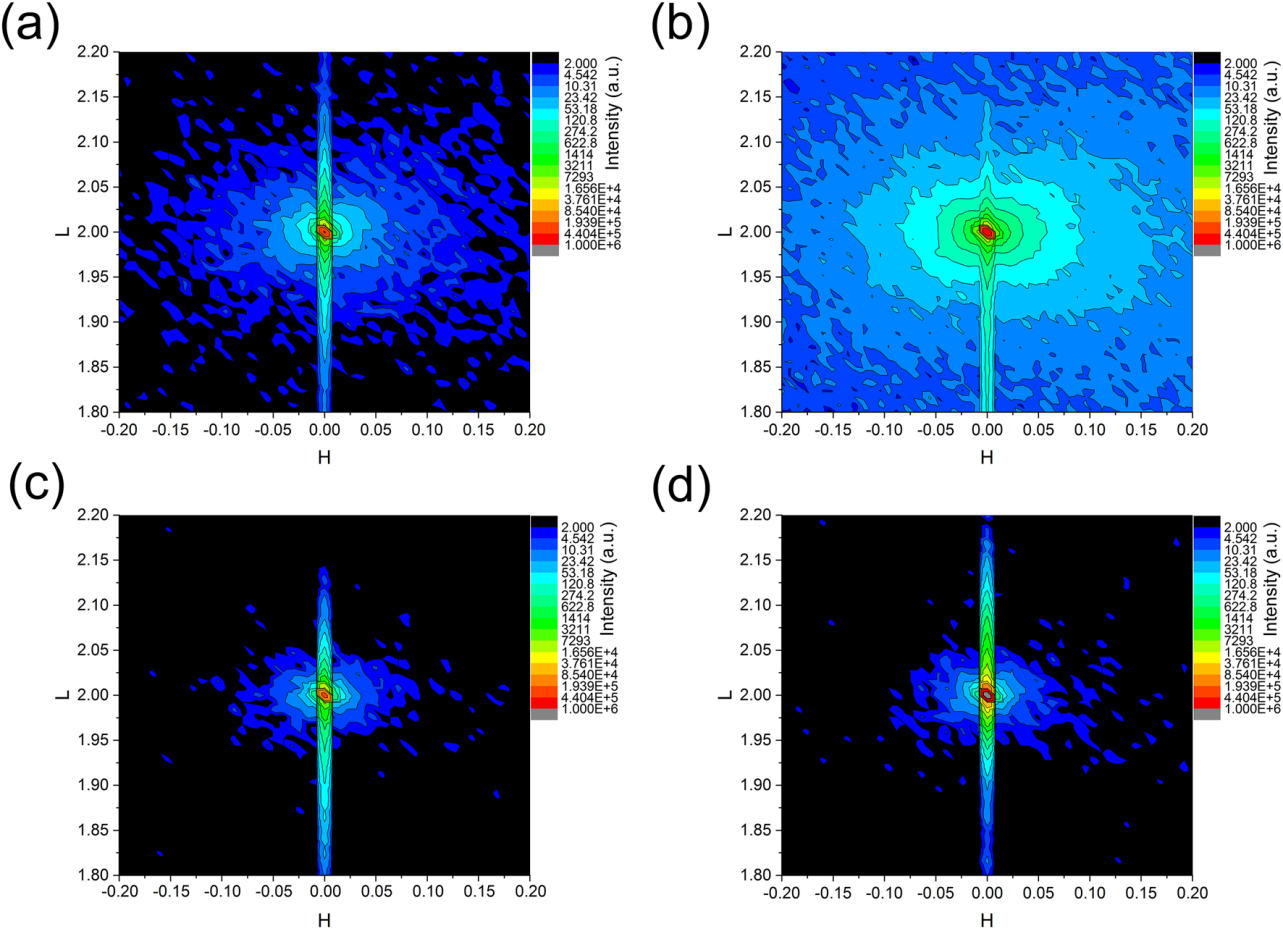
RSMs (reciprocal space mapping) near symmetric 002 reflection of (001)-oriented SrTiO_3_ single crystals. (**a**) RSM of undoped SrTiO_3_ single crystal; (**b**) RSM of 0.5% Nb doped SrTiO_3_ single crystal; (**c,d**) RSMs of undoped and 0.5% Nb doped SrTiO_3_ single crystal which was annealed in a tube furnace in air at the temperature 930 °C for 2 h.

To verify our hypothesis, we performed reciprocal space mapping(RSM)^43^, which is a very effective way to detect the detailed structure of defects among single-crystals^44,45^. Figure 2 shows several RSMs near symmetric (002) reflection of (001)-oriented SrTiO_3_ single crystals with the X-ray wavelength of 1.2387 Å. As can be seen, the diffuse scattering is extending in all directions indicating that most of the defects are point defect^45^. The diffuse scattering is stronger in the direction of q_L_, it is because of the effect of crystal truncation rod (CTR) scattering, which originates from the interface between the crystal surface and vacuum in the out-of-plane direction^46^. The only difference between the samples is the intensity. It is clearly showing that lots of defects appear in crystals of STO with Nb doping, RSM intensity become more scattered, as in comparing in Fig. 2a,b. The intensity of the diffuse scattering around symmetric (002) Bragg reflection is much stronger due to those defects and lattice structure distortion as calculated from rocking curves. Figure 2c,d show the measured RSM for the annealing Nb-free, and 0.5% Nb-doped STO crystal, which indicates that 2 h annealing in air at 930 °C will eliminate those defects effectively (at least around the near surface part due to the small velocity of the transfer atoms in the crystal), even for the doped crystals. In this way, we suggest the annealing is necessary for the STO or doped STO applications in device fabrication or even only for substrate.

**Figure 3:**
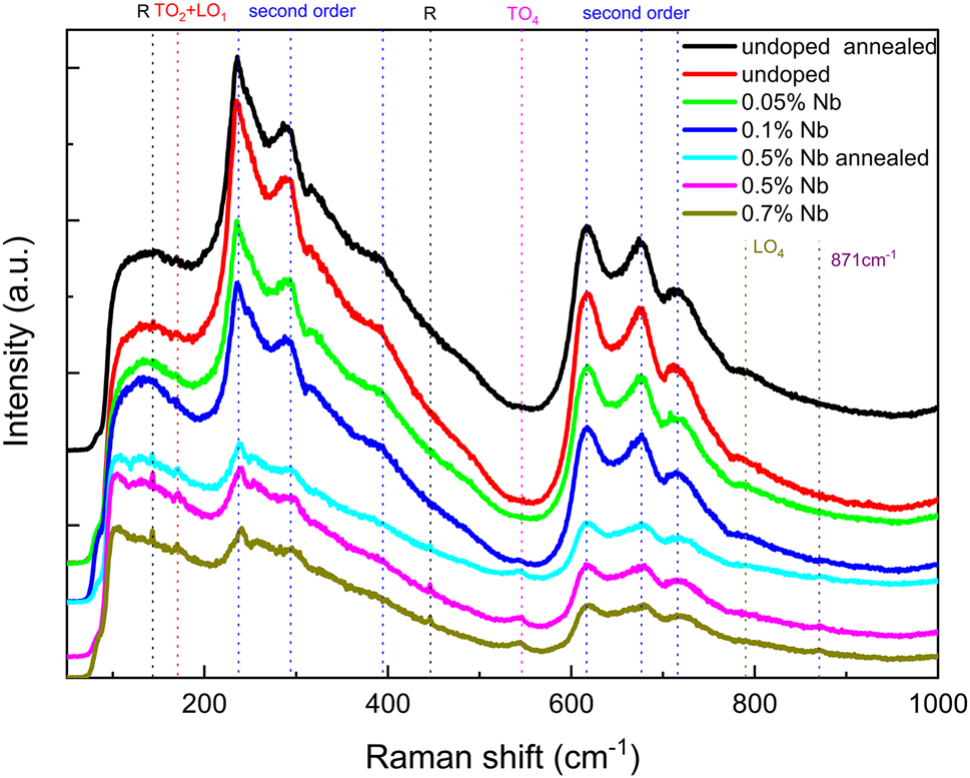
Raman spectra of various Nb-doped SrTiO_3_ single crystals at 110 K.

To observe the soft mode after phase transition we measured Raman spectra of Nb-doped SrTiO_3_ single crystals with varying the temperature. Figure 3 shows the Raman spectra at 110 K. The spectrum of SrTiO_3_ crystal is dominated by the second-order features^47^. Second-order Raman spectra involve the creation or destruction of two phonons, therefore the spectra tend to be continues rather than individual branches in the vibrational spectrum which is the feature of first-order Raman spectra feature. As can be seen, at 110 K, in comparing to the spectrum of SrTiO_3_ at room temperature, first-order peaks assigned to TO_2_ + LO_1_, TO_4_ and LO_4_ have emerge^47^, indicating the local structural symmetry has been broken. For 0.5% and 0.7% Nb-doped SrTiO_3_ single crystals, these first-order peaks remain active at room temperature, meaning more Nb element have break local structural symmetry even at room temperature. It verifies our previous analyses form the XRD, rocking curve and RMS data: Nb doping introduce lattice distortion, lattice expansion, point defects and stacking faults which breaks the local symmetry of the single crystals. Moreover, another very weak scatter peak appeares around 871 cm^−1^, which has been observed in other doped STO and is believed to result from the incorporation of impurity ions in B sites and the distortion of oxygen octahedron^24,48^. As the doping concentration of Nb element gets higher the intensity of second-order features gets weaker and sharp peaks at 144 and 444 cm^−1^ generates at 110 K, indicating the weaken of the cubic structure and the formation of the tetragonal structure. These two peaks are of soft modes called as R^47^, which are zone-edge (R point) phonons becoming Raman active because of the double folding of the Brillouin zone due to structural phase transition at T_AFD_^49^. Here for Nb-doped STO with Nb concentration higher than wt 0.5%, T_AFD_ is clearly higher than 110 K. To determine the exact T_AFD_ of each samples and measure the change of electrical transport property, we performed transport property measurement together with Hall resistivity measurement in the (001) plane.

**Figure 4:**
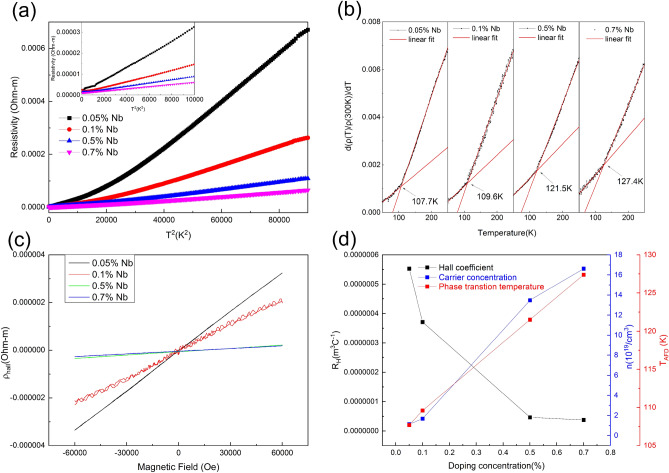
Transport property of Nb doped SrTiO_3_ single crystals. (**a**) Temperature-square dependence of the electrical resistivity; (**b**) The differential curve of the normalized resistivity ρ(T)/ρ(300 K); (**c**) Magnetic Field dependence of the Hall resistivity (ρ_hall_) at 2 K; (**d**) Hall coefficient (R_H_), Carrier concentration (n) and Phase transition temperature (T_AFD_) of various Nb doped SrTiO_3_ single crystals at 2 K.

**Figure 5:**
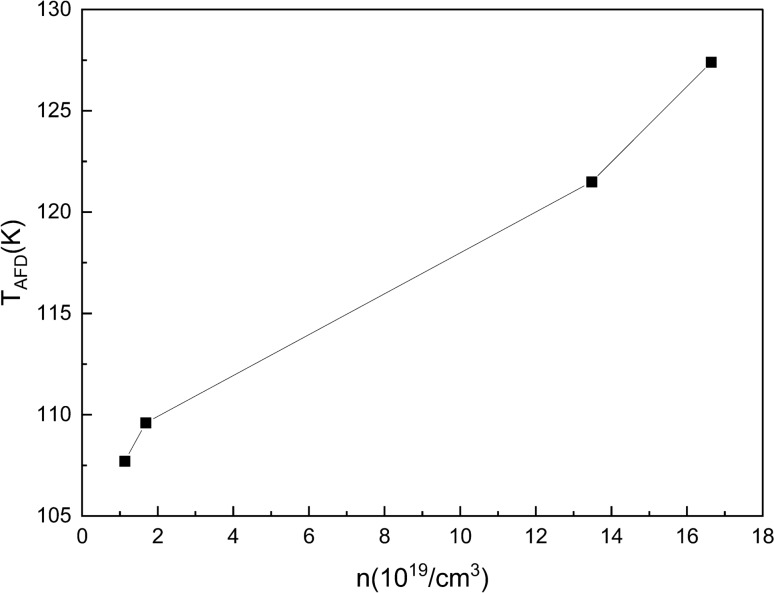
Evolution of the AFD transition temperature with carrier concentration for Nb:STO single crystals.

Nb-free STO behaves as an insulator. Figure 4a shows that the resistivity at room temperature is negatively correlated with the doping concentration and all doped SrTiO_3_ single crystals act as the conducting behavior. It is clear that the resistivity obeys the function $\rho ={\rho }_{0}+A{T}^{2}$ both at high temperature range (above 120 K) and at low temperature range (below 100 K shown in inset of Fig. 4a). However when passing through the phase transition temperature T_AFD_, the parameter A changes. The T-square temperature dependence of resistivity is an indication of a strong electron–electron scattering process. Which is a typical Fermi liquid behavior^50^ in the STO system and widely reported separately at low temperature^51^ and high temperature^39^, yet few work had mention the change of the parameter A, meanwhile the mechanism of this T-square temperature dependence is not settled^19^. We attribute this change of resistivity to the structural phase transition, which also has been mentioned in the mobility fitting of La doped STO^21^. Here we normalized resistivity ρ (T)/ρ (300 K), Fig. 4b shows the differential curve of it. The obvious turning of the curve is marked by black arrow as shown in Fig. 4b, which represent the turning point of the structural phase transition temperature. We linear fit the two part of the curve and the intersection point of the two fitted straight lines marks the structural phase transition. The T_AFD_ of wt 0.05%, 0.1%, 0.5% and 0.7% Nb-doped samples are 107.7 K, 109.6 K, 121.5 K and 127.4 K respectively. It verifies our observation from Raman spectra, that there is an increase of T_AFD_ when an amount of Nb ions are doped into SrTiO_3_ single crystal. Figure 4c shows magnetic Field dependence of the Hall resistivity (ρ_hall_) of various Nb doped SrTiO_3_ single-crystals at 2 K, it shows that the current carrier is electron. Figure 4d shows that the Hall coefficient (R_H_) is negatively correlated with the doping concentration and carrier concentration (n) is linear positive correlated with the doping concentration, the standard error is milli times smaller so that the error bar is negligible compare to the data point. The carrier concentration is in the same magnitude as in Nb-doped STO thin films reported by Ohta et al*.*^52^. Nb-doped STO is a degenerate semiconductor in which the charge carrier concentration depends on the content of defects. Other defect concentration is so little compare to the amount of Nb element (several magnitudes smaller) that no obvious difference can be seen between annealed and other samples (also the annealing process may effect only the near surface region). Figure 4d also shows that T_AFD_ is linear positive correlated with the doping concentration. The correlation between T_AFD_ and carrier concentration is shown in Fig. 5. The T_AFD_ increases with increasing the carrier concentration, i.e., the doping concentration. Our results are the same as those reported by Tao et al*.*^20^ that in Nb:STO T_AFD_ increases with the increasement of the carrier concentration as well as the doping concentration. It is because the disturbance of Nb element weakens the stability of the cubic structure, which is fragilely relied on the balance of three atomic radii of SrTiO_3_. On the other hand, the phase transition temperature (T_AFD_) and the carriers concentration (n) of SrTiO_3_ may be modulated by niobium doping. T_AFD_ increases with increasing the niobium in a rate of about 30 K per (wt%) niobium and n in a rate of about 2.5 $\times$ 10^20^/cm^3^ per (wt%) niobium.

## Conclusions

Strontium titanate single crystals with varying the doping concentration of niobium have been grown and studied in the present. High-resolution X-ray diffraction studies show that as the doping concentration increase the lattice constants increase as well, yet further post-annealing process tend to decrease the defects. The reciprocal space mapping (RSM) near symmetric 002 reflection verifies our suggestion. Raman Spectra of various Nb-doped SrTiO_3_ single crystals at 110 K indicates that there is an increase of phase transition temperature together with local structural symmetry broken when certain amount of Nb ions are doped into SrTiO_3_ single crystal. We observe the change of the electrical transport behavior around the T_AFD_, it can be a new way to quantify the phase transition temperature of Nb-doped SrTiO_3_. Transport property measurement shows that that the current carrier is electron. In addition, carrier concentration (n) is linear positive correlated with the doping concentration as well as the phase transition temperature T_AFD_. We propose that the lattice parameter (c), phase transition temperature (T_AFD_) and the carriers concentration (n) of SrTiO_3_ may be modulated by niobium doping. T_AFD_ increases with increasing the niobium in a rate of about 30 K per (wt%, i.e. niobium element’s weight verses total weight) niobium and n in a rate of about 2.5 $\times$ 10^20^/cm^3^ per (wt%) niobium.

## Data Availability

The datasets generated during and/or analysed during the current study are available from the corresponding author on reasonable request.
